# Comparison of Protein Carbonyl (PCO), Paraoxonase-1 (PON1) and C-Reactive Protein (CRP) as Diagnostic and Prognostic Markers of Septic Inflammation in Dogs

**DOI:** 10.3390/vetsci8060093

**Published:** 2021-05-29

**Authors:** Beatrice Ruggerone, Donatella Scavone, Roberta Troìa, Massimo Giunti, Francesco Dondi, Saverio Paltrinieri

**Affiliations:** 1 Department of Veterinary Medicine, University of Milan, Via Celoria, 10, 20133 Milano, Italy; beatrice.ruggerone@gmail.com (B.R.); donatella.scavone@unimi.it (D.S.); saverio.paltrinieri@unimi.it (S.P.); 2 Veterinary Teaching Hospital, University of Milan, Via dell’Università 6, 26900 Lodi, Italy; 3 Ospedale Veterinario I Portoni Rossi, Via Roma, 57/a, Zola Predosa, 40069 Bologna, Italy; 4 Department of Veterinary Medical Science, Alma Mater Studiorum, University of Bologna, Ozzano dell’Emila (BO), 40064 Bologna, Italy; roberta.troia2@unibo.it (R.T.); f.dondi@unibo.it (F.D.)

**Keywords:** acute phase protein, canine, inflammation, oxidative stress, prognosis, sepsis

## Abstract

Reliable diagnostic and prognostic markers of sepsis are lacking, but essential in veterinary medicine. We aimed to assess the accuracy of C-Reactive Protein (CRP), protein carbonyls (PCO) and paraoxonase-1 (PON1) in differentiating dogs with sepsis from those with sterile inflammation and healthy ones, and predict the outcome in septic dogs. These analytes were retrospectively evaluated at admission in 92 dogs classified into healthy, septic and polytraumatized. Groups were compared using the Kruskal–Wallis test, followed by a Mann–Whitney *U* test to assess differences between survivors and non-survivors. Correlation between analytes was assessed using the Spearman’s test, and their discriminating power was assessed through a Receiver Operating Characteristic (ROC) curve. PON1 and CRP were, respectively, significantly lower and higher in dogs with sepsis compared with polytraumatized and clinically healthy dogs (*p* < 0.001 for both the analytes), and also in dogs with trauma compared with healthy dogs (*p* = 0.011 and *p* = 0.017, respectively). PCO were significantly increased in septic (*p* < 0.001) and polytraumatized (*p* < 0.005) as compared with healthy dogs. PON1 and CRP were, respectively, significantly lower and higher in dogs that died compared with survivors (*p* < 0.001 for both analytes). Ultimately, evaluation of CRP and PON1 at admission seems a reliable support to diagnose sepsis and predict outcomes.

## 1. Introduction

The management of dogs with systemic and severe clinical signs associated with sepsis often represents a challenge in clinical practice: veterinarians should provide reliable information to owners both about the prognosis and the treatment cost estimation. The decision to hospitalize or to euthanize a pet is often strictly correlated to these features. Moreover, the early exclusion of septic processes is pivotal to select those cases on which antibiotic therapy is not necessary or even harmful for the patient. For example, canine pancreatitis is frequently associated with a systemic inflammatory response syndrome (SIRS) and its possible consequences (e.g., acute kidney injury, multi organ dysfunction, disseminated intravascular coagulation) without being related to a septic etiology [1,2,3,4]. In these cases, the administration of antibiotics could modify the gut microbiota, causing additional disabling symptoms (e.g., diarrhea) and weakening the immune system [5,6,7,8]. Moreover, the abuse of antibiotics in patients without septic conditions would amplify the worldwide problem of drug resistance.

Unfortunately, reliable diagnostic and prognostic markers of sepsis are still lacking in veterinary medicine. Little information is available about the potential utility of markers commonly used in human medicine to diagnose or stage sepsis. Several markers have been tested in recent years, including methemoglobin fractions, adiponectin, apolipoprotein A1 [9,10,11] and especially procalcitonin. The latter has an acknowledged relevant role in diagnosing sepsis in people, while some limitations were reported in dogs [12,13]. Specifically, some concern was initially raised about the analytical accuracy of commercially available reagents in measuring canine procalcitonin [14], but a recent report demonstrated its increase in serum of dogs with experimentally induced endotoxemia [15]. However, clinical studies involving septic patients demonstrated a substantial overlap with results recorded in non-septic conditions [16] and its usefulness in practice seems to be associated with its ability to predict organ dysfunction, especially through serial measurements over the course of the disease [17]. Before these recent studies, C reactive protein (CRP) has been widely used as a marker of sepsis in dogs [18,19,20] and remains to date the most used in routine practice. However, CRP may have a low specificity in diagnosing sepsis, because its serum concentration may increase in several non-septic inflammatory or non-inflammatory conditions [21,22,23,24,25,26].

Inflammation is characterized by a sequela of events, mostly associated with the activation of phagocytes, which generates reactive oxygen species (ROS) and other oxidants. This induces an imbalance between oxidants and antioxidant defenses known as oxidative stress, due to the sustained production of oxidative compounds and to the consumption of antioxidants, whose serum concentration decreases [27]. During sepsis, a hyperinflammatory state develops, and the resulting oxidative stress is higher than in non-septic inflammation [28,29,30,31,32]. Paraoxonase-1 (PON1) is a serum enzyme with antioxidant properties. During inflammation, serum PON1 activity decreases, due to both changes in composition and structure of circulating PON1 and decreased hepatic synthesis [33]. Decreases in serum PON1 activity have already been demonstrated in some, but not all, dogs with inflammation [34,35], likely because oxidation is particularly intense only in some types of inflammation or when inflammation is more severe. As a support to this latter hypothesis, several studies postulated that serum PON1 may be a marker of severity of inflammation [9,36,37,38].

The ROS produced during oxidative stress lead to protein oxidation, and in particular, to the oxidation of protein side chains, with the subsequent generation of protein carbonyl (PCO) groups (aldehydes and ketones) [39]. In people, protein carbonylation is the most widely used biomarker for oxidative damage to proteins, since it reflects cellular damage induced by multiple forms of ROS [40]; it has been demonstrated that the concentration of plasma protein carbonyls is significantly higher in septic patients compared with controls [41]. In veterinary medicine, few studies have been developed about protein carbonylation. Validation studies on commercially available ELISA kits are lacking, except for a recent study that demonstrated that antibody-based techniques may reliably detect PCO in canine serum and that PCO values seem to be inversely proportional to PON1 levels in dogs with inflammatory diseases [42]. Vannucchi et al. [43] used PCO along with other markers of oxidation to evaluate the presence and magnitude of oxidative stress associated with pregnancy in healthy dogs, but no significant changes in the concentration of PCO were found, and the used method was not validated in dogs. The study of Escobar et al. [44] assessed plasma PCO both in horses and in dogs with Leishmania without evidence of a significant difference between sick animals and the control group. On the other hand, Zini et al. [45] found higher levels of carbonyls in the erythrocytes of diabetic cats, with a significant decrease after treatment.

For the aforementioned reasons, we hypothesized that dogs with sepsis would exhibit higher CRP and PCO concentration and lower PON1 activity, due to severe inflammation and oxidative stress, compared with dogs with non-septic inflammation, and that these differences could be helpful from a diagnostic, and possibly prognostic, point of view. The aims of this study were: (1) to compare which analyte, among CRP, PCO and PON1, better differentiates dogs with sepsis from those with polytrauma or from healthy dogs; (2) to preliminarily investigate the possible role of CRP, PCO or PON1 in predicting the prognosis of septic dogs, by assessing if results obtained at admission for each analyte may be associated with a negative clinical outcome.

## 2. Materials and Methods

### 2.1. Caseload

This retrospective study was done on 92 serum samples collected from privately owned dogs (37 males, 10 castrated males, 29 females and 16 neutered females) that underwent clinical examination at the University of Bologna and Milan. Samples were stored at the same Institutions at −80 °C for a maximum of 12 months. The median age of the dogs included in the study was 36 months (age range: 1 month–15 years). Thirty-six dogs were mongrels, whereas the other dogs were: German Shepherds (*n* = 8), Golden Retrievers (*n* = 4), Cavalier King Charles Spaniels, Labrador Retrievers, American Staffordshire Terriers, Italian Bloodhounds (*n* = 3 for each breed), Pugs, English Bulldogs, Miniature Poodles, Doberman Pinschers, Maremmano-Abruzzese Sheepdogs, Jack Russell Terriers, Rottweilers (*n* = 2 for each breed), Maltese, Leonberger, Yorkshire Terrier, Bernese Mountain Dog, English Setter, Dachshund, Shetland Sheepdog, Airedale Terrier, Australian Shepherd, Great Dane, Shih Tzu, Cocker Spaniel, Chihuahua, Weimaraner, Italian Pointer, Dogo Argentino, Saluki, Rhodesian Ridgeback, Spanish Greyhound (*n* = 1 for each breed). Dogs were divided into the following three groups:

- Group A, clinically healthy: 35 dogs (12 males, 7 castrated males, 10 females, 6 neutered females; median age 24 months, age range: 6 months–13 years) that were considered healthy on the basis of normal physical examination, history and blood test results;
- Group B, septic: 34 dogs (12 males, 2 castrated males, 12 females, 8 neutered females; median age 60 months; age range: 1 month–15 years) that were considered septic as a result of the presence of symptoms such as abnormal mentation, fever or hypothermia, tachycardia, tachypnoea and of appropriate tests on the basis of the suspected diagnosis (e.g., inflammatory leukogram, cytology consistent with presence of intracellular bacteria, abdomen ultrasound examination, thorax X-rays, positive blood culture) [46];
- Group C, non-septic inflammation: 23 dogs (13 males, 1 castrated male, 7 females, 2 neutered females; median age 24 months; age range: 3 months–15 years) polytraumatized after motor vehicle accidents (*n* = 15) or falls (*n* = 3) or blunt trauma of unknown origin (*n* = 5): in these dogs, sepsis was excluded based on history and collateral tests reported above.

Serum samples collected within two hours since first presentation and before starting any treatment or just after the administration of treatment were included in this study. Then, all the dogs from Groups B and C received appropriate treatments according to the diagnosis. For each sick patient, clinical data and outcome were recorded.

All the samples were collected from client-owned dogs for diagnostic purposes or during routine examinations; an informed consent about the use of residual amounts of samples for research purposes was signed by the owners. Therefore, according to the regulations of our Institution, it was not necessary to require a formal authorization to the Institutional animal care committee (Decision of the institutional committee no. 2/16 dated 15/02/16).

### 2.2. Measurement of PCO, PON1 and CRP

The serum concentration of PCO was measured in 41 samples (15 from Group A, 14 from Group B and 12 from Group C) using a commercially available ELISA kit (Enzo Life science, 3V Chimica, Roma, Italy) following manufacturer’s instructions. The plate was read with an automated plate reader (Dasit multiscan, Dasit, Cornaredo, Milan, Italy) using a wavelength of 450 nm. A regression standard curve was then designed by plotting the lot specific nmoL/mg protein carbonyl concentration of the standards, against their absorbances. The concentration of PCO in each sample was then calculated by interpolating the absorbance of each sample with the standard curve.

The serum activity of PON1 was measured in all the samples included in this study using an automated chemistry analyzer (Cobas Mira, Roche Diagnostics, Basel, Switzerland), as previously described [34,47]. Serum PON1 activity was measured spectrophotometrically using the enzymatic method proposed by [33]: briefly, 6 µL of samples were incubated at 37 °C with 89 µL of distilled water and 100 µL of reaction buffer (glycine buffer 0.05 mM, pH 10.5 containing 1 mM of paraoxon-ethyl, purity > 90%, and 1 mM of CaCl_2_). The rate of hydrolysis of paraoxon to p-nitrophenol was measured by monitoring the increase in absorbance at 405 nm using a molar extinction coefficient of 18.050 L mol^−1^ cm^−1^. The unit of PON1 activity expressed as U/mL is defined as 1 nmol of p-nitrophenol formed per minute under the assay conditions.

The serum concentration of CRP was measured in 87 serum samples (35 from Group A, 32 from Group B and 20 from Group C) using the automated analyzer BT3500 (Biotecnica Instruments SPA, Roma, Italy) using an immunoturbidimetric method provided by the manufacturer of the analyzer.

### 2.3. Statistical Analysis

Statistical analysis was performed in an Excel spreadsheet using a specific software (Analyse-it, Analyse-it Software Ltd., Leeds, UK). Statistical differences were set for *p* < 0.05.

The mortality rates recorded in the two pathologic groups were compared to each other using a Pearson chi-square test. Results regarding PCO, PON1 and CRP were compared in each group of dogs using the Kruskal–Wallis test, followed by a Mann–Whitney *U* test to assess the differences between single groups. The Mann–Whitney *U* test was used also to compare the results obtained at admission from animals that died with those that survived. These comparisons were performed either on the whole caseload or on sick dogs (Groups B and C), in order to draw information more relevant to the clinical application of the test.

A Spearman’s correlation test was used to correlate the results of PCO, PON1 and CRP in the 36 samples on which all the analytes were measured.

In order to assess the discriminating power of the three analytes in detecting dogs with sepsis or with a poor outcome and to establish the optimal diagnostic cut-off, for each numerical value recorded in the study (operating point), we classified as true or false positive the dogs with or without sepsis or the dogs that died or not, that had PCO or CRP values higher and PON1 values lower than each operating point, and as true or false negative the dogs with or without sepsis or the dogs that died or not that had PCO or CRP values lower and PON1 values higher than each operating point. Sensitivity and specificity and the positive likelihood ratio were calculated for each analyte and for each operating point, using standard formulae [48,49], and receiver operating characteristic (ROC) curves were designed by plotting sensitivity versus (1–specificity) [48]. The Youden index (i.e., the operating point that maximizes the difference between true positives and false positives) and the operating points characterized by the highest LR+ and by absolute specificity were then calculated [50]. Additionally, the evaluation of sensitivity and specificity and ROC curve analyses were performed first by also including the control group and then excluding the control group.

## 3. Results

### 3.1. Group Composition

The mortality rate was significantly lower (*p* = 0.027) in the non-septic group (3/23; 13.0%) than in the septic group (14/34; 41.2%). No significant differences were found regarding the age (*p* = 0.150) or the proportion of male or female dogs (*p* = 0.211) among the three groups.

### 3.2. Group Comparison and ROC Curve Analyses for the Diagnosis of Sepsis

**Figure 1:**
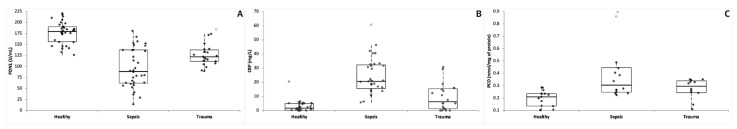
Serum PON1 activity (**A**) and concentration of CRP (**B**) and PCO (**C**) in clinically healthy dogs, in dogs with sepsis and in those with polytrauma. The boxes indicate the I-III interquartile range (IQR), the horizontal black line indicates the median values, whiskers extend to further observation within quartile I minus 1.5 × IQR or to further observation within quartile III plus 1.5 × IQR. Black dots indicate the results that are not classified as outliers. White dots indicate near outliers (values exceeding the third quartile ± (1.5 × IQR)) and grey dots indicate far outliers (values exceeding the third quartile ± (3.0 × IQR)).

A significant difference among groups was found for the serum concentrations of PCO and CRP as well as for PON1 activity (*p* < 0.001 for all the analytes, Table 1, Figure 1).

**Figure 2:**
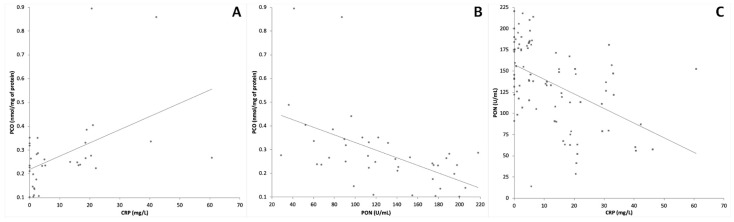
Correlations between CRP and PCO (**A**), PON1 and PCO (**B**), and CRP and PON1 (**C**).

In particular, the concentration of PCO was significantly higher in dogs with sepsis and in dogs with trauma than in clinically healthy dogs, but no significant differences were found between dogs with trauma or sepsis. Conversely, the serum concentration of CRP was significantly higher in dogs with sepsis than in dogs with trauma and in clinically healthy dogs; a significant difference was also found between dogs with trauma and clinically healthy dogs. Similarly, PON1 activity was significantly lower in dogs with sepsis than in dogs with trauma and in clinically healthy dogs, and a significant difference was found also between dogs with trauma and clinically healthy dogs. The same differences were found when only results from dogs with sepsis or trauma were compared to each other, although the level of significance was lower than in the previous comparison for PON1 (*p* < 0.05). On the whole caseload, PON1 was negatively correlated either with PCO (*p* < 0.001, *r* = −0.594) or with CRP (*p* < 0.001, *r* = −0.510) while PCO and CRP were positively correlated to each other (*p* = 0.007, *r* = 0.440) (Figure 2).

**Figure 3:**
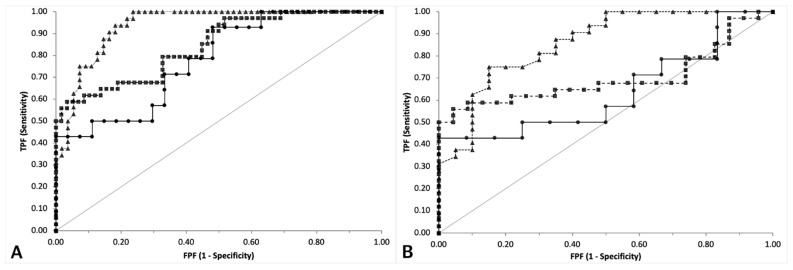
Comparison of ROC curves to support a diagnosis of sepsis designed for PCO (black circles, continuous line), CRP (black triangles, dotted line) and PON1 (black squares, dashed line). The graph on the left (**A**) includes the results of the three groups of dogs, the graph on the right (**B**) includes only the results of dogs with sepsis or trauma. The grey line indicates the line of no discrimination. TPF = true positive fraction; FPF = false positive fraction.

The ROC curve analysis (Figure 3) on the whole caseload demonstrated that all the three analytes had a discriminating power for sepsis (*p* < 0.001 compared with the line of no discrimination) (Table 2). However, the area under the curve (AUC) of PON1 and of CRP were significantly higher than the AUC of PCO, but not significantly different to each other (*p* = 0.990).

Results were substantially similar when the comparison above was repeated excluding clinically healthy dogs, despite all the AUCs were slightly lower than those recorded in the previous comparison and only the ROC curve of PON1 and of CRP, but not that of PCO, had a discriminating power (*p* = 0.001, *p* < 0.001 and *p* = 0.115, respectively). As in the previous comparison, the AUCs of PON1 and CRP and were significantly higher than that of PCO (*p* = 0.034 and *p* = 0.035, respectively), but not significantly different to each other (*p* = 0.928).

CRP had the highest Youden index, although the cut-off to discriminate septic vs. non-septic dogs was higher when clinically healthy dogs were excluded from the analysis, as expected. The same occurred for PCO and PON1, whose optimal cut-offs were, respectively, higher and lower when clinically healthy dogs were excluded from the analysis. Conversely, all the tests had the highest LR^+^ or absolute specificity at similar cut-offs regardless of the presence or absence of clinically healthy dogs, but the LR+ was obviously lower when clinically healthy dogs were excluded.

### 3.3. Group Comparison and ROC Curve Analyses Based on the Outcome

**Figure 4:**
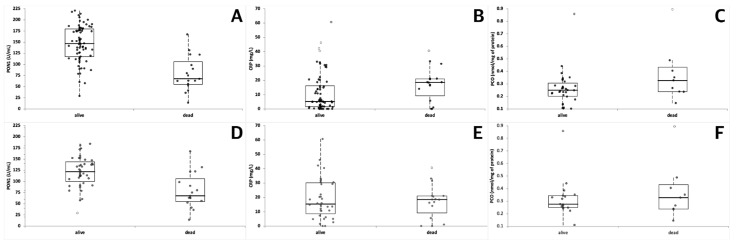
Serum activity of PON1 (**A**,**D**) and concentration of CRP (**B**,**E**) and PCO (**C**,**F**) in dogs that survived or died during the follow up. The analysis was performed either on the whole caseload (upper graphs) or after exclusion of clinically healthy dogs (lower graphs). The boxes indicate the I-III interquartile range (IQR), the horizontal black line indicates the median values, whiskers extend to further observation within quartile I minus 1.5 × IQR or to further observation within quartile III plus 1.5 × IQR. Black dots indicate the results that are not classified as outliers. White dots indicate near outliers (values exceeding the third quartile ± (1.5 × IQR)), and grey dots indicate far outliers (values exceeding the third quartile ± (3.0 × IQR)).

Overall, PON1 activity was significantly lower in dogs that died compared with dogs that survived (Table 3, Figure 4).

Similarly, the concentration of CRP was significantly different in dogs that died compared with dogs that survived, although with a lower level of significance. Conversely, despite the P value being very close to the level of statistical significance (*p* = 0.078), the concentration of PCO was not significantly different in dogs that survived compared with dogs that died. Excluding healthy controls from the analysis, PON1 activity was still significantly lower in dogs that died compared with dogs that survived, and the concentration of PCO was still not significantly different (*p* = 0.726) in dogs that survived compared with dogs that died. On the contrary, the concentration of CRP showed no significant differences (*p* = 0.897) in dogs that died compared with dogs that survived.

**Figure 5:**
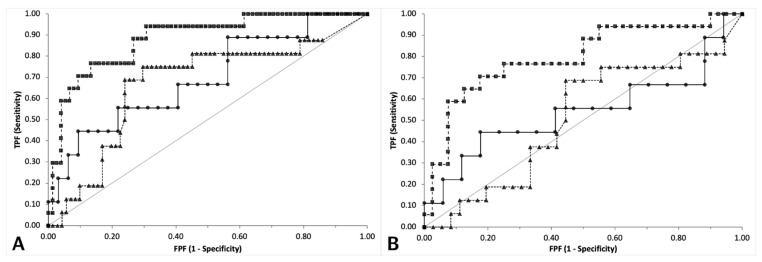
Comparison of ROC curves to support a poor outcome designed for PCO (black circles, continuous line), CRP (black triangles, dotted line) and PON1 (black squares, dashed line). The graph on the left (**A**) includes the results of the three groups of dogs, the graph on the right (**B**) includes only the results of dogs with sepsis or trauma. The grey line indicates the line of no discrimination. TPF = true positive fraction; FPF = false positive fraction.

Based on the ROC curve analysis on the whole caseload (Table 4, Figure 5), only the AUC of PON1 had a discriminating power (*p* < 0.001 compared with the line of no discrimination) and was significantly higher than the AUC of CRP (*p* < 0.001).

The AUC of PCO and CRP did not significantly differ from the line of no discrimination although the P value of PCO was close to the level of significance (*p* = 0.068). No significant differences were found between the AUCs of PCO and CRP (*p* = 0.218) or of PCO and PON1 (*p* = 0.062). The exclusion of clinically healthy dogs provided the same results: although the AUCs were lower for all the analytes, only the AUC of PON1 was significantly different from the line of no discrimination (*p* < 0.001) and significantly higher than the AUC of CRP (*p* < 0.001). The AUC of PCO and CRP did not significantly differ from the line of no discrimination and were not significantly different to each other (*p* = 0.339) as well as no significant differences were found between the AUCs of PCO and PON1 (*p* = 0.069).

The highest Youden index was found for PON1, although the cut-off to discriminate dead vs. alive dogs was lower when clinically healthy dogs were excluded from the analysis, as expected. The same occurred for PCO and CRP whose optimal cut-offs were higher when clinically healthy dogs are excluded than when clinically healthy dogs are included in the analysis. All the tests had the highest LR^+^ or absolute specificity at similar cut-offs, independent of the presence or absence of clinically healthy dogs. However, the LR+ were obviously lower when clinically healthy dogs were excluded and the maximum specificity was detected only at very high values of CRP or PCO or at very low values of PON1.

## 4. Discussion

In several species, it has already been proved that sepsis induces an acute phase reaction, that in turn is characterized by an increase of acute phase proteins such as CRP [18,51,52], and by oxidative phenomena. The latter may induce protein oxidation, leading to the increase of the serum concentration of PCO [40,41] and to the decrease of antioxidant compounds, including the enzyme paraoxonase-1 [33,53,54]. Different studies are available about the utility of CRP to distinguish dogs with sepsis from dogs with sterile inflammation [55,56,57,58,59], but only a recent study demonstrated that PON1 may be useful to discriminate dogs with sepsis from dogs with non-septic inflammation, although ultimately, CRP and albumin may better predict the outcome [60] and no information is available about the utility of PCO in dogs as diagnostic or prognostic marker, differently from human medicine [28,29,32,54,61,62,63].

In the case where a possible diagnostic or prognostic role could be demonstrated, the evaluation of CRP, PCO and/or PON1 could be recommended as a tool to better focus on the severity of the inflammatory status in the enrolled patients. To this aim, two different approaches have been followed. First, the comparison of results, the evaluation of sensitivity and specificity and the ROC curves analysis was performed by including the whole caseload, composed by both sick and clinically healthy dogs, in order to simulate the use of the markers for screening purposes independently of a pre-test probability of disease. Then, the comparison was performed excluding the control group, to simulate the use of the markers for diagnostic purposes in animals on which a diagnosis of a condition potentially associated with sepsis has already been formulated.

This study confirmed the possible role of CRP in supporting a clinical diagnosis of sepsis, as already evidenced by previous studies [18,19,20], since, regardless of the inclusion or the exclusion of clinically healthy dogs, the highest concentration of CRP was found in dogs with sepsis and the ROC curve analysis demonstrated that CRP had the highest AUC. However, CRP concentration was increased also in dogs with trauma, that likely had an hyperacute, although non-infectious, inflammation. This confirmed that increases of CRP may occur in different inflammatory conditions and are thus not specific for sepsis [18,21,22,23,24,25,26] and slightly decreased the diagnostic performance of CRP when the test was applied only on sick dogs. A similar trend was observed for PON1, that was significantly lower in septic dogs than in the other two groups and decreased also in dogs with polytrauma compared with controls. The AUC of the ROC curve referred to PON1, in agreement with what was reported by [60], was slightly lower, but not significantly different from that of CRP, although, also in this case, the diagnostic performance of the test decreased, but remained acceptable, when only sick dogs were considered.

From this perspective and compared with PCO, CRP and PON1 seem to be better markers to differentiate the three conditions considered in this study. This hypothesis is confirmed both by the group comparisons and by the analysis of the ROC curves. Additionally, the LR^+^ of CRP and, to a lesser extent, of PON1 were notably higher than that of PCO either on the whole caseload or when the tests were applied to discriminate groups of sick dogs. However, the concentration of CRP characterized by absolute specificity was relatively high, and the PON1 activity characterized by absolute specificity was relatively low, compared with the upper and lower limits of the reference interval, respectively. Hence, moderate increases of CRP or moderate decreases of PON1 may not differentiate dogs with sepsis from dogs with non-septic conditions.

Moreover, the comparison of results from dogs that died during the follow up with survivors suggests that the decrease of PON1 and, to a lesser extent, the increase of CRP may predict a poorer outcome. More specifically, the analyte that better differentiated dogs with a poor outcome in clinical settings on which the likelihood of death is high, was found to be PON1 activity. In fact, the concentration of CRP did not show significant differences between survivors and non-survivors when healthy controls, which are obviously not supposed to die, were excluded from the analysis. Ultimately, when data were examined on the whole caseload, results were in contrast with those of [64], who demonstrated that CRP levels did not differ between survivors and non-survivors, but were in agreement with the study of [65], in which CRP was considered a potentially useful clinical marker for the presence and resolution of systemic inflammation induced by infectious agents in dogs. Our results also agree with those of [60], although these authors reported increased CRP as a better predictor of negative outcome than decreased PON1. These discrepancies may be explained by the different composition of the caseload, especially considering the lack of a uniform consensus regarding the inclusion criteria of septic patients among existing studies. This aspect remains troublesome and partially impairs a fair comparison of biomarkers between patients enrolled with different criteria, as demonstrated by a superior role of PON1 compared with CRP in predicting the outcome after exclusion of clinically healthy dogs. This clinical scenario, however, is much more adherent to what may happen in practice, when prognostic information is needed in dogs with a high pre-test probability of disease. To reduce discrepancies between different studies about sepsis diagnosis and to support the clinical work, further research about diagnostic and prognostic markers of sepsis should indeed be encouraged. In any case, based on the analysis of LR+ and cut-off values characterized by maximum specificity, it should be stressed that either PON1 or CRP may be excellent indicators of a poor prognosis only if recorded values are, respectively, extremely low or high compared with the refence intervals. Independently of all the comments above or of the inclusion/exclusion of clinically healthy dogs, PCO seems not to predict the outcome in dogs.

A limitation of this study is that the presence of oxidation was not confirmed by the measurement of oxidants such as reactive oxygen species, or other indirect reliable markers of oxidation, such as thiobarbituric acid reactive substances or malondialdehyde [66,67]. However, the study was focused on markers that can be easily used in routine practice and that are known to be associated with oxidation. This association is further confirmed by the negative correlation found, as expected, between PCO and PON1. Ultimately, the comparison of results of CRP, PCO and PON1 confirm the hypothesis that in dogs with sepsis, oxidative phenomena are stronger than in dogs with non-septic inflammation, as already demonstrated for people [68,69,70,71,72]. This hypothesis is supported also by the positive correlation between CRP and PCO and by the negative correlation between PCO and the other two markers. However, the magnitude of oxidation seems to affect the kinetic of the two analytes in different ways. More specifically, oxidation of proteins, which is responsible for the increased concentration of PCO, seems to occur in any inflammatory condition able to determine an increase of CRP, including those not associated with infectious agents. On the contrary, the decrease of PON1 activity, which depends on a more complex mechanism involving decreased hepatic production and displacement of circulating PON1 from oxidized lipoproteins [34], seems to occur only when inflammation is more severe, as it usually happens in sepsis. Independent of the possible mechanisms by which oxidation differently affects the two compounds, based on our results, PCO cannot be considered reliable markers to distinguish dogs with sepsis from dogs with non-septic inflammation or predict the outcome, while PON1 and CRP may be considered adequate markers to support a clinical diagnosis of sepsis and provide prognostic information on the possible outcome. A second limitation of the study was the lack of clinical information about severity of disease and organ dysfunction for many dogs. These aspects would have been remarkable to evaluate, but unfortunately, as a retrospective study, the retrieval of such information was frequently troublesome. Finally, another limitation was the lack of repeated sampling during the follow up. This would be particularly important to assess a possible prognostic role of PCO not detected by a single measurement at admission. Fluctuations over time of PCO concentrations may have provided prognostic information, as demonstrated for other markers with a limited prognostic role at admission, such as calprotectin, procalcitonin, CRP or PON1 [17,36,73,74,75,76].

In our opinion, measurement of CRP and PON1 could be an advisable tool to support a clinical diagnosis of sepsis and predict the outcome in routine practice. In clinical practice, the use of CRP as marker of inflammation is already established and widespread, while evaluation of PON1 is to date quite limited to the research field. However, PON1 measurement is quite inexpensive and, given that interest about its applications in veterinary medicine has lately been raised, its measurement may become easily accessible for clinical practice in the future years.

## 5. Conclusions

In conclusion, these results confirm that oxidation is present during inflammatory conditions and especially in sepsis and support the role of CRP and PON1 in confirming a clinical diagnosis of sepsis. Moreover, the measurement of PON1 and CRP at admission, but not of PCO, may predict the possible outcome of dogs with sepsis. Future studies on a larger caseload or on serial measurements of these markers over time may provide additional information about their prognostic role in septic dogs.

## Figures and Tables

**Table 1:** Mean ± SD, median (between parenthesis) and min-max range of PCO, CRP and PON1 in dogs with sepsis, trauma and clinically healthy dogs.

Dogs	PON1 (U/mL)	CRP (mg/L)	PCO (nmol/mg of Protein)
Sepsis	96.4 ± 44.4 (88.7) *** ^††† (‡)^ 14.1–180.9	24.22 ± 12.47 (20.60) *** ^††† (‡‡‡)^ 5.66–60.70	0.40 ± 0.22 (0.31) *** 0.22–0.89
Trauma	126.6 ± 25.1 (111.5) *** 89.6–184.4	9.46 ± 9.36 (6.17) * 0.00–30.60	0.27 ± 0.08 (0.30) ** 0.11–0.35
Clinically healthy	176.8 ± 24.4 (179.5) 126.0–220.6	2.88 ± 3.76 (1.72) 0.00–20.50	0.20 ± 0.06 (0.21) 0.10–0.29

*** *p* < 0.001 vs. clinically healthy; ** *p* < 0.01 vs. clinically healthy; * *p* < 0.05 vs. clinically healthy; ^†††^ *p* < 0.001 vs. trauma; ^‡‡‡^ *p* < 0.001 vs. trauma when only dogs with sepsis and trauma were compared to each other; ^‡^ *p* < 0.05 vs. trauma when only dogs with sepsis and trauma were compared to each other.

**Table 2:** Area under the ROC curves (AUCs), Youden index and operating points characterized by the highest LR+ and by absolute specificity for PON1, CRP and PCO to support a diagnosis of sepsis, calculated either including or excluding clinically healthy dogs from the analysis.

Statistical Data	Whole Population			Only Sick Dogs		
	PON1 (U/mL)	CRP (mg/L)	PCO (nmol/mg of Protein)	PON1 (U/mL)	CRP (mg/L)	PCO (nmol/mg of Protein)
AUC (%)	95 (87–104) *	95 (91–100) *	78 (63–93)	90 (76–104) *	90 (79–100) *	63 (41–86)
Youden index	98.6 (Y: 0.554)	5.64 (Y: 0.764)	0.23 (Y: 0.447)	91.5 (Y: 0.515)	15.90 (Y: 0.600)	0.35 (Y:0.429)
Max LR+	90.9 (LR^+^ 30.7)	29.20 (LR^+^ 20.6)	0.35 (LR^+^ 11.6)	91.1 (LR^+^ 12.8)	29.20 (LR^+^ 7.5)	0.35 (LR^+^ 5.1)
100% Sp	89.6	30.60	0.35	89.6	30.30	0.35

* *p* < 0.05 compared with PCO.

**Table 3:** Mean ± SD, median (between parenthesis) and min-max range of PCO, CRP and PON1 in dogs that died and dogs that survived, both including and excluding clinically healthy dogs.

Dogs	PON1 (U/mL)	CRP (mg/L)	PCO (nmol/mg of Protein)
Dead	79.1 ± 39.0 (67.6) *** ^†††^ 14.1–167.4; *n* = 17	17.36 ± 11.70 (18.50) * 0.00–40.60; *n* = 16	0.37 ± 0.22 (0.33) 0.15–0.89; *n* = 9
Alive (all the groups)	146.6 ± 40.9 (146.7) 28.9–220.6; *n* = 74	11.25 ± 13.24 (5.20) 0.00–60.70; *n* = 70	0.26 ± 0.14 (0.25) 0.10–0.86; *n* = 32
Alive (only dogs with sepsis or trauma)	121.1 ± 34.3 (121.7) 14.1–106.4; *n* = 40	19.07 ± 14.22 (15.35) 0.00–60.70; *n* = 36	0.32 ± 0.16 (0.28) 0.11–0.86; *n* = 17

*** *p* < 0.001 vs. alive (all the groups); * *p* < 0.05 vs. alive (all the groups); ^†††^ *p* < 0.001 vs. alive (only dogs with sepsis or trauma).

**Table 4:** AUCs, Youden index and operating points characterized by the highest LR+ and by absolute specificity for PON1, CRP and PCO to support a poor outcome, calculated either including or excluding clinically healthy dogs from the analysis.

Statistical Data	Whole Population			Only Sick Dogs		
	PON1 (U/mL)	CRP (mg/L)	PCO (nmol/mg of Protein)	PON1 (U/mL)	CRP (mg/L)	PCO (nmol/mg of Protein)
AUC (%)	90 *** (82–99)	54 (38–70)	70 (48–90)	82 *** (68–96)	39 (22–57)	54 (27–82)
Youden index	132.7 (Y:0.635)	13.90 (Y:0.454)	0.35 (Y: 0.351)	90.9 (Y: 0.531)	15.90 (Y:0.243)	0.35 (Y:0.268)
Max LR+	57.7 (LR^+^ 22.1)	15.90 (LR^+^ 2.9)	0.44 (LR^+^ 7.1)	57.7 (LR^+^ 11.8)	15.90 (LR^+^ 1.5)	0.44 (LR^+^ 3.8)
100% Sp	28.9	60.70	0.86	28.9	60.70	0.86

*** vs. CRP.

## Data Availability

Data is contained within the article (tables and dot plots).
